# A Model of Dendritic Cell Therapy for Melanoma

**DOI:** 10.3389/fonc.2013.00056

**Published:** 2013-03-19

**Authors:** Lisette DePillis, Angela Gallegos, Ami Radunskaya

**Affiliations:** ^1^Department of Mathematics, Harvey Mudd CollegeClaremont, CA, USA; ^2^Department of Mathematics, Loyola Marymount UniversityLos Angeles, CA, USA; ^3^Department of Mathematics, Pomona College, Claremont CollegesClaremont, CA, USA

**Keywords:** mathematical model, cancer, immunotherapy, melanoma, dendritic cell vaccine

## Abstract

Dendritic cells are a promising immunotherapy tool for boosting an individual’s antigen-specific immune response to cancer. We develop a mathematical model using differential and delay-differential equations to describe the interactions between dendritic cells, effector-immune cells, and tumor cells. We account for the trafficking of immune cells between lymph, blood, and tumor compartments. Our model reflects experimental results both for dendritic cell trafficking and for immune suppression of tumor growth in mice. In addition, *in silico* experiments suggest more effective immunotherapy treatment protocols can be achieved by modifying dose location and schedule. A sensitivity analysis of the model reveals which patient-specific parameters have the greatest impact on treatment efficacy.

## 1 Introduction

A promising immunotherapy approach to treating certain cancers involves the use of dendritic cells (DCs). DCs are part of the antigen-specific (adaptive) immune response and function as antigen-presenting cells. Immature DCs are derived in the bone marrow and reside in peripheral tissues. Upon encountering pathogen, DCs begin to mature, and travel to the lymphoid organs where they stimulate differentiation and maturation of cytotoxic T lymphocytes (CTLs). Some of these activated CTLs then travel to the infected tissue to form part of the adaptive immune response, while others become memory cells that are ready to mount a rapid response in case of a rechallenge by the pathogen.

Previous studies have established the efficacy of dendritic cell treatments for tumors in the murine system (DeMatos et al., 1998; Fields et al., 1998; Lee et al., 2007; Yamaguchi et al., 2007; Shinagawa et al., 2008). In these studies, DCs have been shown both to inhibit the growth of nascent tumors and to provide a memory response to previously encountered antigen. In the clinic, researchers have been able to extract immature dendritic cells from patients, culture them *ex vivo*, and load them with tumor antigens to create an individual-based vaccine that can boost a patient’s response against their own cancerous cells (Pilon-Thomas et al., 2004; Taquet et al., 2008). The success of clinical trials of DC vaccines has resulted in the recent FDA approval of the first cancer vaccine for prostate cancer (Cheever, 2011). Despite promising clinical responses in vaccine trials, it remains difficult to predict which patients will actually respond to these vaccines and why (Trefzer et al., 2005; Boon et al., 2006). Mathematical models of DC therapy can provide insight into the mechanisms driving the kinetics of the immune response that may lead to these disparate patient responses.

Cell trafficking is an important aspect of the DC-mediated immune response. DCs must travel from the tumor to the peripheral lymph organs via the blood, and activated CTLs must travel from the lymph organs back to the tumor. Ludewig et al. (2004) have developed a model describing DC and CTL trafficking in mice. The model includes activated and memory CTLs to capture both the immediate and long-term effect of DC injections. The DC trafficking model of Ludewig et al. was carefully calibrated using experimental data from murine studies.

In this paper we present an extension and modification of the model in Ludewig et al. (2004). Our extended model includes a tumor compartment to allow for analysis of various DC treatments and their effect on tumor growth, as well as the long-term behavior of the system. We find relevant model parameters using the data collected by Lee et al. (2007) describing tumor growth in response to varying levels of DC injections. We compare model simulations of various DC doses, injection sites, and dose times. We include a comparison to a prophylactic dosing schedule presented by Preynat-Seauve et al. (2007).

## 2 The Model

The compartment model proposed by Ludewig et al. (2004) includes dendritic cells, activated CTLs, and memory CTLs. Our extended model includes tumor cells in addition to these immune cell populations. Adding a tumor compartment requires the determination of tumor-immune system parameters such as immune cell trafficking rates to and from the tumor, effector cell deactivation rates by tumor cells, effector cell death rates, intrinsic tumor growth rates, and tumor cell kill rates by effector cells. We note that this compartment model does not account for the geometry of the system. In particular, it does not explicitly incorporate the distance between the spleen and the tumor. However, in murine models, the transit times between compartments are small relative to the tumor growth time scale, so this simplification is reasonable. In this section we describe the processes included in the mathematical model.

### 2.1 Model definition

Our model consists of three compartments: the spleen, the blood, and the tumor. Dendritic cells and active effector cells can move between the blood and spleen compartments, and between the blood and tumor compartments. We assume that memory effector cells can move between the spleen and the blood compartments. The system is not conservative: all types of cells are cleared through the blood, immune cells are created in response to the presence of tumor, and tumor cells grow according to a logistic growth law.


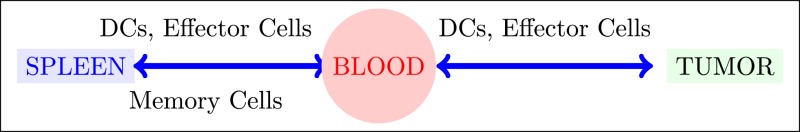


The nine state variables in our model are:

- *D_blood_*, the number of dendritic cells in the blood compartment;
- *D_spleen_*, the number of dendritic cells in the spleen compartment;
- $E_{blood}^{a},$ the number of activated CTLs in the blood compartment;
- $E_{spleen}^{a},$ the number of activated CTLs in the spleen compartment;
- $E_{blood}^{m},$ the number of memory CTLs in the blood compartment;
- $E_{spleen}^{m},$ the number of memory CTLs in the spleen compartment;
- $E_{tumor}^{a},$ the number of activated CTLs in the tumor compartment;
- *T*, the number of tumor cells;
- *D_tumor_*, the number of dendritic cells in the tumor compartment, the tumor-infiltrating DCs.

We present the system of nine differential equations in groupings representing the blood, spleen, and tumor compartments. The model parameters are described in detail in Table A1 in Appendix.

#### 2.1.1 Blood compartment

The equations describing DC and CTL flow in the blood are given by:

(1) $$\begin{array}{lll} \frac{d}{dt}D_{blood} & =-\mu_{B}D_{blood}+\mu_{TB}D_{tumor}+v_{blood}\left(t\right) & \end{array}$$

(2) $$\begin{array}{lll} \frac{d}{dt}E_{blood}^{a} & =\mu_{SB}\left(D_{spleen}\right)E_{spleen}^{a}-\mu_{BB}E_{blood}^{a} & \end{array}$$

(3) $$\begin{array}{lll} \frac{d}{dt}E_{blood}^{m} & =\mu_{SB}\left(D_{spleen}\right)E_{spleen}^{m}-\mu_{BB}E_{blood}^{m} & \end{array}$$

where, as holds throughout the model, the *μ* parameters represent flow rates between compartments. We include the “trapping” term from Ludewig et al. (2004) which describes the observed phenomenon of activated CTLs being held back in the spleen in the presence of DCs:

$$\begin{array}{rcll} \mu_{SB}\left(D_{spleen}\right) & =\mu_{SB}^{*}+\frac{\Delta \mu }{1+\frac{D_{Spleen}}{\theta_{shut}}}, & & \\ \Delta \mu & =\mu_{SB}^{Normal}-\mu_{SB}^{*}. & & \end{array}$$

The function *v_blood_*(*t*) allows us to model injections of DCs into the blood. For example, two doses of 7 × 10^5^ each given on Day 0 and Day 7 could be described by the function:

(4) $$v_{blood}\left(t\right)=\left\{\begin{array}{cc} \frac{7\times 10^{5}}{1∕48},\; & t\in \left(0,1∕48\right)\cup \left(7,7+1∕48\right),\\ 0\; & \text{otherwise}\text{.} \end{array}\right.$$

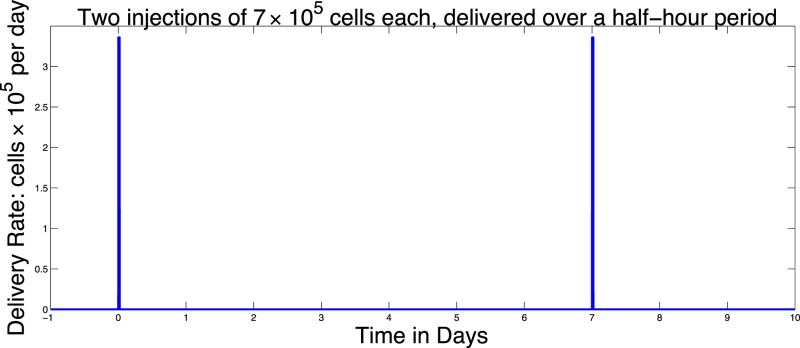


#### 2.1.2 Spleen compartment

The differential equations for the spleen compartment describe interaction, death, and recruitment of DCs and CTL. The equations include one delay which represents the synaptic connection time: the contact time required between DCs and effector cells in the spleen before proliferation can begin. The other interactions we account for in the system do not involve a required contact time and thus are modeled without delay. The dynamics of the populations in the spleen are described by:

(5) $$\begin{array}{llll} \frac{d}{dt}D_{spleen} & =Max\;D\left(1-e^{\left(\frac{-\mu_{BS}D_{blood}}{Max\;D}\right)}\right) & & \\ & \;-a_{D}D_{spleen}-b_{DE}E_{spleen}^{a}D_{spleen} & \end{array}$$

(6) $$\begin{array}{llll} \frac{d}{dt}E_{spleen}^{a} & =\mu_{BSE}E_{blood}^{a}-\mu_{SB}\left(D_{spleen}\right)E_{spleen}^{a}+b_{a}D_{spleen}E_{spleen}^{m} & & \\ & \;+a_{E_{a}S}\left(DC_{on}E_{naive}-E_{spleen}^{a}\right)-r_{am}E_{spleen}^{a} & \\ & \;+b_{p}\frac{D_{spleen}\left(t-\tau_{D}\right)E_{spleen}^{a}\left(t-\tau_{D}\right)}{\theta_{D}+D_{spleen}\left(t-\tau_{D}\right)} & & \end{array}$$

(7) $$\begin{array}{llll} \frac{d}{dt}E_{spleen}^{m} & =r_{am}E_{spleen}^{a}-\left(a_{E_{m}}+b_{a}D_{spleen}+\mu_{SB}\left(D_{spleen}\right)\right)E_{spleen}^{m} & & \\ & \;+\mu_{BSE}E_{blood}^{m}. & \end{array}$$

Note that the term in equation (6),

$$DC_{on}=\left\{\begin{array}{cc} 0\; & \text{if }D_{spleen}\left(t\right)=0\\ 1\; & \text{if }D_{spleen}\left(t\right)>0. \end{array}\right.$$

indicates that we do not allow for new CTLs in the absence of DCs. Thus, the populations we model only exist due to the presence of tumor and mature DCs.

The first term in equation (5) reflects our assumption that there is a maximum rate at which mature DCs can enter the spleen. This is in agreement with observations that DCs cannot enter the spleen at an unlimited rate. Based on a range of values for the maximum rate we have set *MaxD* to 400 (cells per hour), reflecting the parameter fit obtained with the data from Lee et al. (2007) and Preynat-Seauve et al. (2007). As noted above, in equation (6) the final term introduces a delay, *τ*, into the system that reflects the synaptic connection time. Mathematically, this delay introduces more complexity into the system, especially regarding the stability analysis of the equilibria (see *Stability Analysis* below).

#### 2.1.3 Tumor compartment

The tumor compartment contains activated effector CTLs, DCs, and tumor cells. The interactions of these populations within the tumor are described by:

(8) $$\begin{array}{lll} \frac{d}{dt}E_{tumor}^{a} & =\mu_{BTE}\left(T\right)E_{blood}^{a}-a_{E_{a}T}E_{tumor}^{a}-cE_{tumor}^{a}T, & \end{array}$$

(9) $$\begin{array}{lll} \frac{d}{dt}T\; & =rT\left(1-\frac{T}{k}\right)-DT. & \end{array}$$

(10) $$\begin{array}{lll} \frac{d}{dt}D_{tumor} & =\frac{mT}{q+T}-\left(\mu_{TB}+a_{D}\right)D_{tumor}+v_{tumor}\left(t\right) & \end{array}$$

where

$$\mu_{BTE}\left(T\right)=\mu_{BB}\left(T∕\left(\alpha +T\right)\right),$$

and

(11) $$D=d\frac{{\left(\frac{E_{tumor}^{a}}{T}\right)}^{l}}{s+{\left(\frac{E_{tumor}^{a}}{T}\right)}^{l}.}$$

The function *v_tumor_*(*t*) is similar to *v_blood_*(*t*) in the blood compartment, allowing us to inject DCs intratumorally in order to compare treatment protocols.

**Figure 1:**
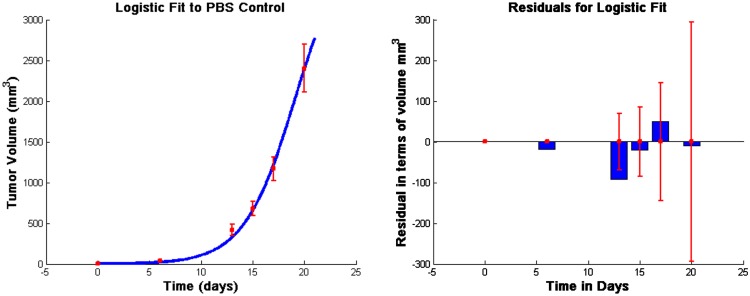
**Fits to data from Lee et al. (2007) and corresponding residuals**. The vertical bars in the graph on the left are the error bars from the experimental data, and the solid line is the outcome of the model simulation using the parameters that minimize the distance to the median of those data. In the graph on the right, the thin vertical bars are the same error bars from the data, and the solid rectangles show the “residuals,” that is, the distances between the simulated outcomes and the data. The estimated parameter values are given in Table A1 in Appendix.

Note that in equation (9), tumor growth is fit to a logistic function as in previous models (de Pillis and Radunskaya, 2003; de Pillis et al., 2005, 2007, 2009; Cappuccio et al., 2006). The behavior of this particular model is robust to the choice of growth function, for example a Gompertz growth law gives similar results. However, we choose the logistic law since it provides a good fit to the experimental data we are using for model calibration (See Figure 1). Cytolysis of tumor cells by activated CTLs [equations (9) and (11)] is a ratio-dependent kill term introduced in de Pillis and Radunskaya (2003). Experimental results from Diefenbach et al. (2001) support ratio-dependent, antigen-specific killing, and the term has been employed to success in previous models (de Pillis and Radunskaya, 2003; de Pillis et al., 2005, 2007, 2009). The importance of tumor-infiltrating dendritic cells has been demonstrated in several studies. See, for example, Preynat-Seauve et al. (2007). We allow DCs in the tumor to increase as a saturation-limited function of the size of the tumor population.

With this model we simulate a variety of treatment scenarios, including those investigated in Lee et al. (2007) and Preynat-Seauve et al. (2007). The model offers insight into how best to harness the tumor controlling potential of DCs.

## 3 Results

### 3.1 Parameter determination

All unknown parameters were fit to data from Lee et al. (2007) using a Nelder–Mead least-squares algorithm. The data in Lee et al. (2007) were collected from C57BL/6 female mice who were subcutaneously inoculated with 5 × 10^5^ B16F10 melanoma cells. We take this as day 0 for the purpose of fitting unknown parameters so that we may use 5 × 10^5^ tumor cells as an initial condition. Injections of 1 × 10^5^, 7 × 10^5^, or 21 × 10^5^ DCs were given at days 6, 8, and 10, following inoculation with tumor cells. Additionally, a control group was injected with PBS according to the same schedule (Lee et al., 2007). See Figure 1.

### 3.2 Equilibria and stability analysis

In order to determine the long-term behavior of the system, we find the equilibria and determine their stability.

#### 3.2.1 Determination of the equilibria

The system has multiple equilibrium values, determined by setting equations (1–10) to zero. One solution to this system is the zero, or disease-free, equilibrium. To find the remaining non-zero equilibria, we first write all the state variables at equilibrium as functions of *T*, then search for the values of *T* that solve all equations simultaneously. We use asterisks to denote the value of the variables at equilibrium. Therefore, if there exists a non-zero value T* that satisfies equation (9), we can use equation (8) to obtain

(12) $$E_{tumor}^{a*}={\left\{\frac{s{\left(T^{*}\right)}^{l}\left(k-T^{*}\right)}{T^{*}-k\left(1-d∕r\right)}\right\}}^{1∕l}.$$

The value for $D_{tumor}^{*}$ can also be found in terms of T* using equation (10):

(13) $$D_{tumor}^{*}=\frac{mT^{*}}{q+T^{*}}\frac{1}{\mu_{TB}+a_{D}},$$

where we have replaced *DC_death_* with its assumed constant value *a_D_*.

Given $E_{tumor}^{a*}$, we can use equation (8) to determine the equilibrium value of the active effector cells in the blood:

(14) $$E_{blood}^{a*}=\frac{\left(a_{E_{a}T}+cT^{*}\right)\left(\alpha +T^{*}\right)}{\mu_{BB}T^{*}}E_{tumor}^{a*}.$$

We use equations (13) and (1) to obtain $D_{blood}^{*}$ in terms of T*:

$$D_{blood}^{*}=\frac{\mu_{TB}D_{tumor}^{*}}{\mu_{B}}=\frac{\mu_{TB}}{\mu_{B}}\left(\frac{1}{\mu_{TB}+a_{D}}\right)\frac{mT^{*}}{q+T^{*}}.$$

Equation (3) gives an expression for $E_{blood}^{m*}$ in terms of $E_{spleen}^{m*}$,

$$E_{blood}^{m*}=\frac{\mu_{SB}\left(D_{spleen}\right)}{\mu_{BB}}E_{spleen}^{m*}.$$

Turning to the spleen compartment, we have:

$$E_{spleen}^{a*}=\frac{\mu_{BB}}{\mu_{SB}\left(D_{spleen}\right)}E_{blood}^{a*}.$$

Using (14) and (12), this gives $E_{spleen}^{a*}$ in terms of T*. According to equation (5), knowing $D_{blood}^{*}$ allows determination of $D_{spleen}^{*}$. Using equation (6) results in the following quadratic equation for $D_{spleen}^{*}$:

(15) $$\begin{array}{ll} 0 & =-\theta_{shut}\left(\mu_{SB}^{*}+\Delta \mu \right)D_{in}\left\{\theta_{shut}\mu_{SB}^{*}a_{D}+\Delta \mu \theta_{shut}a_{D}\right.\\ & \;\left.+\theta_{shut}b_{DE}\mu_{BB}E_{Blood}^{a*}-\mu_{SB}^{*}D_{in}\right\}D_{spleen}^{*}\;\\ & \;\left\{\mu_{SB}^{*}a_{D}+b_{DE}E_{Blood}^{a*}\mu_{BB}\right\}{\left(D_{spleen}^{*}\right)}^{2}, \end{array}$$

where

$$D_{in}=Max\;D\left\{1-exp\left(\frac{-\mu_{BS}D_{blood}}{Max\;D}\right)\right\}.$$

Solving this quadratic equation yields two different, relevant equilibrium values for $D_{spleen}^{*}$. From equation (7) we get a value for $E_{spleen}^{m*}$ for each value of $D_{spleen}^{*}$:

$$E_{spleen}^{m*}=\frac{r_{am}E_{spleen}^{a*}}{a_{E_{m}}+b_{a}D_{spleen}^{*}+\mu_{SB}\left(D_{spleen}^{*}\right)\left\{1-\mu_{BSE}∕\mu_{BB}\right\}}.$$

Finally, from equation (6), the roots of the following function, expressible in terms of one variable, T*, yield equilibrium values for T*.

(16) $$\begin{array}{l} Z(T^{*})=\mu_{BSE}E_{blood}^{a*}-\mu_{SB}(D_{spleen}^{a*})E_{spleen}^{a*}+b_{a}D_{spleen}^{*}E_{spleen}^{m*}\\ \;+a_{E_{a}S}(DC_{on}E_{naive}-E_{spleen}^{a*})-r_{am}E_{spleen}^{a*}\\ \;+b_{p}\frac{D_{spleen}^{*}\;E_{spleen}^{a*}}{\theta_{D}+D_{spleen}^{*}}. \end{array}$$

**Figure 2:**
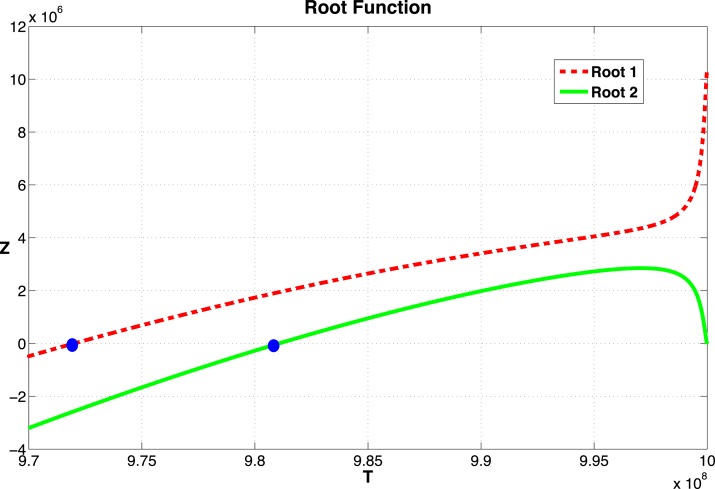
**Graph of the function *Z* given in equation (16)**. The two zeros, marked by dots, correspond to equilibrium values of *T*. The function becomes complex as *T* → 10^9^. The two curves correspond to the two roots of equation (15).

From equation (12), we see that as long as the values of T*, and thus the roots of (16), lie between *k*(1 − *d*/*r*) and *k*, a non-zero equilibrium state exists. Recall that *d*, *r*, and *k* are the parameters that represent the tumor cell kill, intrinsic growth rates, and tumor carrying capacity, respectively. The function *Z*(*T*) is plotted in Figure 2 for the parameter set given in Table A1 in Appendix.

#### 3.2.2 Stability of the equilibrium points

A stability analysis of the system of delay equations (1–10) can be carried out by analyzing the linear approximation to the system at an equilibrium point. Since the term $D\left(T,E_{tumor}^{a}\right),$ given in equation (11), is not differentiable at (0, 0), the system of DEs is not differentiable and, hence, has no linear approximation at the origin. Although we cannot use the linearization in this case, we do have numerical simulations that indicate that the tumor free equilibrium is initially unstable, but gains stability as the value of *d*, the immune strength parameter, is increased (see Figure 9). There is ongoing investigation of the analytical nature of the stability of the disease-free equilibrium.

At other equilibria, the linearization is given by two matrices of partial derivatives, *J*_0_ and *J_τ_*. To simplify the notation, we denote the nine state variables as *x*_1_ through *x*_9_ and the delayed state variables as *z_i_*(*t*) = *x_i_*(*t* − *τ*). If the rate of change of *x_i_* is denoted by:

$$\frac{dx_{i}}{dt}=F_{i}\left(x_{1},\ldots x_{9},z_{1},\ldots z_{9}\right)$$

then the entries of the Jacobians are:

$$J_{0}\left(i,j\right)=\frac{\partial F_{i}}{\partial x_{j}},\;J_{\tau }\left(i,j\right)=\frac{\partial F_{i}}{\partial z_{j}}$$

The formulas for the entries of *J*_0_ and *J_τ_* are given in Appendix B.

The eigenvalues of the derivative matrices at an equilibrium, **E** can be determined by finding the roots of the characteristic polynomial *P*(λ, *τ*), where *P* is defined by:

$$P\left(\lambda ,\tau \right)=det\left(J_{0}\left(\text{E}\right)+e^{-\lambda \tau }J_{\tau }\left(\text{E}\right)-\lambda I\right)$$

**Figure 3:**
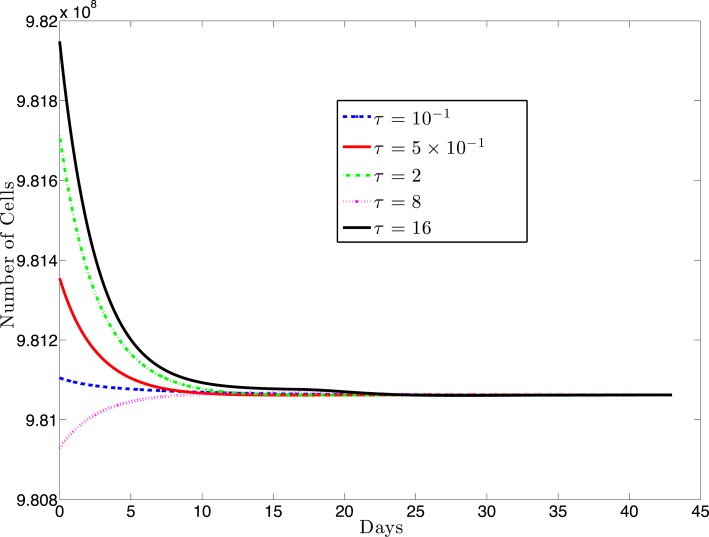
**This figure presents a sampling of several simulations for various values of the delay, τ and several different initial conditions (IC)**. The solid black line (topmost curve) shows a simulation with initial value 9.82 × 10^8^ with τ = 16. The other curves (from top to bottom) are the results of simulations using (τ, IC) pairs: (2, 9.817 × 10^8^), (0.5, 9.835 × 10^8^), (0.1, 9.811 × 10^8^), (8, 9.8 × 10^8^). For each value of τ, we simulated several initial conditions ranging between 9.80 × 10^8^ and 9.82 × 10^8^ (other simulations not shown) and, in each case, the cell populations approached the same equilibrium value.

For most systems of delay-differential equations, determining the roots of the characteristic polynomial is a non-trivial process. Given our equations and particular parameter set, there exist two positive equilibrium values of T* (see Figure 2). However, only one of these values result in positive (biologically relevant) equilibrium values for all other state variables (see Appendix B). For this biologically relevant equilibrium, in the non-delay (or *τ* = 0) case, all eigenvalues of the derivative matrix *J*_0_ have negative real part (see Appendix B). Because this is equivalent to the non-delay case, we know that when *τ* = 0, the non-trivial equilibrium is stable. It is possible for an equilibrium to change stability as the delay increases from zero. In our case numerical simulations suggest that this equilibrium maintains its stability even for large values of the delay, *τ* (see Figure 3).

### 3.3 Calibration and validation of DC effect on tumor growth

In this section we discuss the validation of the model. Starting with parameter values estimated in Ludewig et al. (2004), we then calibrate DC and CTL dynamics against the data provided by the experiments in Lee et al. (2007). In the discussion section we present a number of numerical experiments in which we explore the difference between intravenous and intratumoral DC injections, as well as modifications of the dose timings with hypothetically improved treatment schedules.

Experiments carried out in Lee et al. (2007) give tumor growth data, both in the presence and absence of DC treatment. We fit our intrinsic tumor growth parameters to the PBS melanoma growth data provided by Lee et al. (2007). Since specific trafficking parameters to and from the tumor have not been measured, we used the tumor growth data provided to infer the parameter values needed for the tumor compartment DC and CTL dynamics.

In Lee et al. (2007), groups of three 6–8-week-old female C57BL/6 mice were challenged with 5 × 10^5^ B16F10 melanoma cells on day 1, then treated with DC injections starting on days 6, 8, and 10. In separate experiments, DC doses of size 1 × 10^5^, 7 × 10^5^, and 21 × 10^5^ were administered. Lee et al. point out that the largest DC dose is most effective at slowing tumor growth. In fact, according to Lee et al., the largest dose regimen of 21 × 10^5^ DCs injected three times provided up to 41% tumor growth suppression as compared to the control mice. Survival time for these mice was increased by approximately 60%. We note that, even with the most aggressive DC treatment attempted, tumor growth was not completely suppressed.

**Figure 4:**
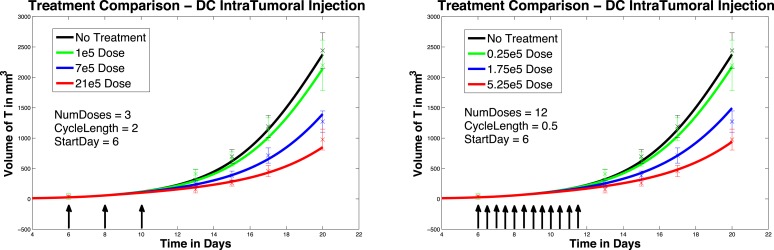
**Fractionated dosing comparison**. Intratumoral injections. Compare original DC dosing schedule (left panel) to hypothetical fractionated dosing schedule (right panel).

In Figure 4, left panel, we see the change in tumor volume over 20 days, and compare tumor growth with no DC treatment to growth with varying levels of DC treatment. Simulated DC doses are 1 × 10^5^, 7 × 10^5^, and 21 × 10^5^, reflecting the laboratory experiments of Lee et al. (2007). After a tumor challenge of 5 × 10^5^ B16F10 melanoma cells on day 1, DC injections of the specified doses were then given intratumorally on days 6, 8, and 10. The simulation results fall well within the data ranges provided by Lee et al. (2007).

## 4 Discussion

In Section 3 we validated and calibrated the model, and analyzed the long-term behavior of the system. We are now in a position to explore hypothetical treatment variations. In this section, we discuss the effects of varying treatment protocols, and possible implications for patients. In Section 4.1, we compare intratumoral DC injections to hypothetical intravenous DC injections. We will see that when injecting the smallest dose of DCs, hypothetical intravenous DC injections are more effective at suppressing tumor growth than are DCs injected directly into the tumor. However, intratumoral injections are more effective than intravenous injections when the highest DC dose is used. In Section 4.2, we explore the effect of modifying dose timings. We will see that fractionated doses that are administered intravenously delay tumor growth significantly. We will see that earlier treatment initiation also helps suppress tumor growth, but more so with intratumoral injections. Up to this point, we have found ways to slow tumor growth by varying dose timing and location, but have not been able to completely eliminate a tumor. In Section 4.3, we explore the effects of prophylactic DC dosing. We find that prophylactic DC dosing actually allows us to eliminate a tumor under the right circumstances. We will see that as long as the CTL immune response is sufficiently strong, as reflected by the immune strength parameter *d*, a tumor that is introduced after DCs are injected can be completely suppressed.

### 4.1 Intratumoral versus intravenous treatment

We first compare the effect of treatment at two different injection sites. In the work of Preynat-Seauve et al. (2007) DC trafficking resulting from different injection sites was compared. They observed that there is a “trapping effect” within the tumor: DCs injected intratumorally do not reach the lymph nodes in significant numbers, indicating that the DCs are “trapped” for a time within the tumor. Note that this trapping is a different phenomenon from the one described in Section 2.1 which referred to activated CTLs being held back in the spleen in the presence of DCs. Preynat-Seauve et al. observed that subcutaneous DC injections resulted in DCs getting to the lymph nodes in greater numbers. In our numerical experiments, we compare intratumoral DC injections (as was done in the Lee et al. (2007) experiments) to intravenous DC injections. We note that intravenous injections and subcutaneous injections are not equivalent, but both approaches do avoid the trapping effect of intratumoral injections.

**Figure 5:**
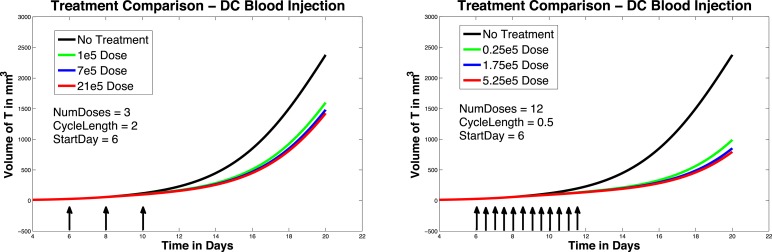
**Fractionated dosing comparison**. Intravenous injections. Compare original DC dosing schedule (left panel) to hypothetical fractionated dosing schedule (right panel). 5 × 10^5^ B16F10 melanoma cells on day 1, the original dosing calls for DC injections of 7 × 10^5^, and 21 × 10^5^ administered every other day on days 6, 8, 10 (pictured in graphs 0.25 × 10^5^, 1.75 × 10^5^, and 5.25 × 10^5^ twice a day on days 6, 7, 8, 9, 10, 11.

In Figure 5, left panel, we see the effects on melanoma growth of hypothetical intravenous DC injections over a 20 day period. Simulated DC doses of size 1 × 10^5^, 7 × 10^5^, and 21 × 10^5^ are administered. The no-treatment tumor growth case is also included for comparison. After a tumor challenge of 5 × 10^5^ B16F10 melanoma cells on day 1, DC injections of the specified doses were then given on days 6, 8, and 10.

It is interesting to note that in these simulations, all three intravenous dose responses appear to be nearly equally effective. There is little difference between low-dose and high-dose intravenous injection outcomes, while there is a significant difference between low-dose and high-dose intratumoral injection outcomes. In addition, when we compare Figure 5, left panel, to Figure 4, left panel, we see that all three intravenous doses control the tumor growth about as effectively as the mid-sized 7 × 10^5^ intratumoral dose (that is, the intravenous doses are all more effective than the lowest tumor dose, but less effective than the highest tumor dose). The reason for this result can be explained mathematically by the presence of the *MaxD* term in the model, equation (5). This term limits the rate at which DCs can enter the spleen, which in turn limits how saturated with DCs the spleen can get. Consequently, this limited inflow rate works against any treatment that attempts to send DCs into the spleen too quickly. *MaxD* term limits DC inflow into the spleen, the total number of DCs in the spleen over *MaxD* limit. In the case of the low-dose injections, the DCs injected intravenously all enter the spleen, since their entry rate is not limited by *MaxD*, while the low intratumoral dose suffers some DC loss from the tumor. This can explain why the low-dose intravenous injection is more effective than the low-dose intratumoral injection. However, in the high-dose injection cases, the number of DCs entering the spleen from the high-dose intravenous treatment is being limited by the *MaxD* inflow ceiling. On the other hand, the high-dose intratumorally injected DCs enter the spleen more slowly, so even though there is still loss from the tumor, a greater total number of DCs remain in the spleen longer than in the case of the high-dose intravenous injection.

As discussed earlier, the model does not explicitly account for the distance between the spleen and the tumor. If we were to extend the model so that it could apply to human subjects, these distances could vary significantly between individuals. One possible model extension would incorporate the effect of transit times with a partial differential equation that includes a distance *L* along which cells diffuse and convect.

### 4.2 Modified dosing

In this section we explore the hypothetical effect of modifying dose timings. We first investigate the effect of administering the same total medication over a 20 day time frame, but with more frequent injections; that is, a *fractionated* dosing schedule. The original dosing schedule starts on day 6, and administers a total of three doses spaced apart by 2 days. The hypothetical fractionated dosing schedule we explore also starts on day 6, but administers doses twice a day at 1/4 the original dose.

In Figure 4, we compare simulated melanoma growth in response to DCs administered according to the original protocol with a hypothetical fractionated dosing schedule. Injections are given intratumorally. The no-treatment tumor growth case is also included for comparison. After a tumor challenge of 5 × 10^5^ B16F10 melanoma cells on day 1, the original dosing calls for DC injections of 1 × 10^5^, 7 × 10^5^, and 21 × 10^5^ administered every other day on days 6, 8, 10. The hypothetical fractionated schedule administers doses of 0.25 × 10^5^, 1.75 × 10^5^, and 5.25 × 10^5^ twice a day on days 6, 7, 8, 9, 10, 11. The total DC treatment administered is the same in the original and fractionated dosing scenarios. It is clear that the fractionated schedule does not improve outcomes in the case of intratumoral injections.

In Figure 5, we again compare the original DC dosing schedule to a fractionated dosing schedule, but we now use intravenous injections. As before, after a tumor challenge of 5 × 10^5^ B16F10 melanoma cells on day 1, the original treatment schedule calls for DCs of doses 1 × 10^5^, 7 × 10^5^, and 21 × 10^5^ given every other day on days 6, 8, 10. The hypothetical fractionated schedule administers doses of 0.25 × 10^5^, 1.75 × 10^5^, and 5.25 × 10^5^ twice a day on days 6, 7, 8, 9, 10, 11. The total amount of DC administered is the same in both scenarios. The simulations highlight that although the fractionated schedule does not improve outcomes in the case of intratumoral injections, greater tumor control is observed when the fractionated treatment is administered intravenously. Although tumor growth is slowed with the intravenously dosed fractionated schedule, it is not completely controlled, and the tumor still eventually grows.

**Figure 6:**
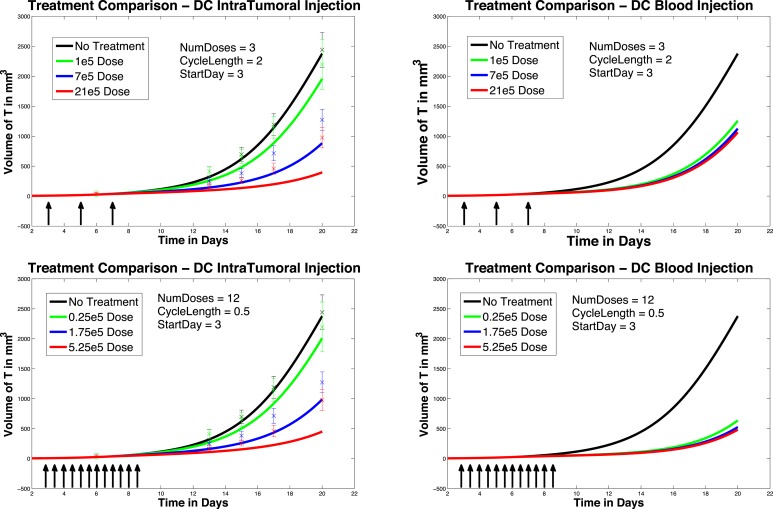
**Early treatment initiation: start day 3**. Original dosing (top row) and fractionated dosing (bottom row) compared. 5 × 10^5^ B16F10 melanoma cells on day 1, DC injections of 7 × 10^5^, and 21 × 10^5^ are administered both intratumorally and intravenously 0.25 × 10^5^, 1.75 × 10^5^, and 5.25 × 10^5^ twice a day on days 3, 4, 5, 6, 7, 8. Treatment is given both intratumorally (left column) and intravenously (right column).

**Figure 7:**
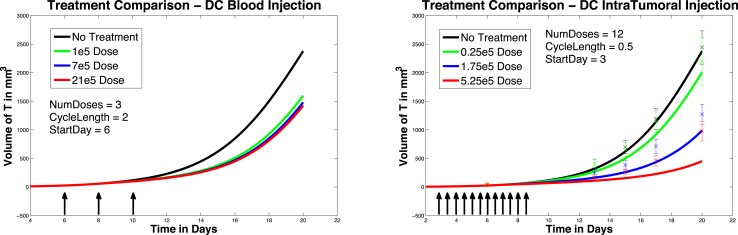
**Compare original to earlier treatment initiation, intravenous, non-fractionated dosing**. Treatment start day 6 (left) compared to start day 3 (right).

We next investigate the effect of starting the DC treatment regimen earlier than day 6. In this case, we compare fractionated DC doses both intratumorally and intravenously, but with treatment initiated on day 3 instead of day 6. The experimental outcomes are pictured in Figure 6. After a tumor challenge of 5 × 10^5^ B16F10 melanoma cells on day 1, DC injections of 1 × 10^5^, 7 × 10^5^, and 21 × 10^5^ are administered both intratumorally and intravenously on days 3, 5, 7 (pictured in graphs in the top row of Figure 6). The hypothetical fractionated schedule (pictured in the second row of Figure 6) administers doses of 0.25 × 10^5^, 1.75 × 10^5^, and 5.25 × 10^5^ twice a day on days 3, 4, 5, 6, 7, 8. The total DC treatment administered is the same in all scenarios. We see that in both the intratumoral and intravenous dosing cases, tumor growth is slowed when treatment starts on day 3. However, as before, fractionating the intratumoral doses does not have much effect (bottom left panel), but does slow tumor growth even further when administered intravenously (bottom right panel). Interestingly, the earlier start day has less effect when administered intravenously than it does when administered intratumorally, as can be seen in Figure 7. Here we compare non-fractionated intravenous dosing starting on day 6 (left panel) and on day 3 (right panel). Initial values and doses follow the original schedule. The result with fractionated dosing is similar, but is not pictured. We see that there is some improvement with the early start intravenous dosing, but the improvement is not as large as the improvement seen with the intratumoral doses started on day 3, as pictured in the left column of Figure 6.

### 4.3 Prophylactic vaccination

So far, we have been investigating the responses of our system to DC treatment *after* a tumor challenge. However, DC treatments have also been considered to have potential as prophylactic vaccines. For example, the work of Preynat-Seauve et al. (2007) details a variety of studies on tumor growth in mice inoculated with DC treatments *prior to* a tumor challenge. Although our model has not been constructed specifically to investigate preventative vaccination, we did see some interesting results when simulating such treatment. In one of the experiments from Preynat-Seauve et al. (2007), DC cells from tumors were cultivated and injected into B6C3F1 mice. Vaccination was performed twice, once weekly, with 10^5^ tumor-infiltrating dendritic cells. The authors state that this number corresponds to the total number of CD11c+ cells recovered from a single 1 cm-diameter tumor. Two weeks after the last injection, mice were challenged with 2 × 10^5^ melanoma cells (either K1735 or B16F10). According to the study, vaccinated mice were protected for 22 days, whereas naive mice succumbed to the tumor challenge.

**Figure 8:**
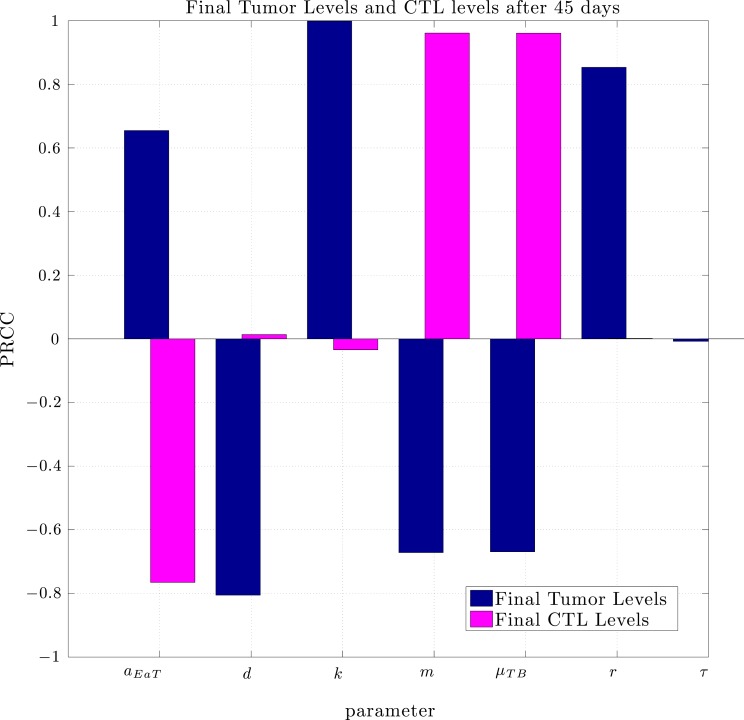
**Partially Ranked Correlation Coefficients (PRCC) for two outcomes: final tumor size, and final CTL levels**. Parameters with negative PRCCs relative to final tumor levels are negatively correlated with tumor growth. Increasing such parameters would be beneficial to the patient. An increase in parameters with positive PRCCs relative to final tumor levels, or with negative PRCCs relative to final CTL levels could be harmful to the patient.

Using the same parameter values we determined through fitting to the data in Lee et al. (2007), a simulation of pre-vaccination with mature DCs showed no particular benefit. In order to determine the sensitivity of the system to a change in parameters we used the Latin Hypercube sampling method described in Blower and Dowlatabadi (1994) to compare simulations with 50,000 randomly generated parameter sets. The effect of a change in parameter values on tumor size and CTL levels after 45 days was quantified by calculating the partially ranked correlation coefficients for each parameter that showed a monotonic relationship to the outcomes. The results are shown in Figure 8.

**Figure 9:**
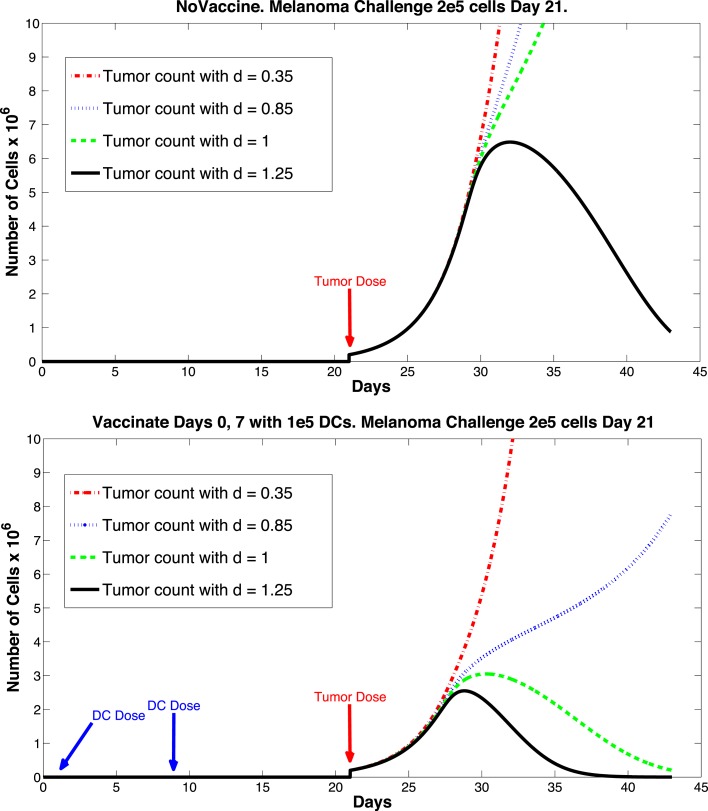
**Prophylactic vaccination and the effect of varying immune strength parameter *d***. Top panel, no vaccine. Bottom panel, vaccinate with DC treatments, days 0 and 7, 1 × 10^5^ DCs per dose. Tumor challenge on day 21, with 2 × 10^5^ tumor cells. Dosing follows Preynat-Seauve et al. (2007) experiment. With *d* = 1, tumor is controlled as a result of vaccination.

The sensitivity analysis indicates that tumor levels are sensitive to the parameters *d*, *m*, and *μ_TB_*. Since *d*, the fractional tumor kill rate by CTLs, has the potential to be manipulated through treatments (*c.f*. Chakraborty et al., 2003), we suggest that this parameter might play an important role in the vaccine’s effectiveness. In subsequent simulations, when *d* was increased we observed a protective effect of prophylactic vaccination with DCs. In Figure 9 we see tumor growth both without and with DC vaccination with *d* = 0.35, 0.85, 1.0, and 1.25. The results for *d* = 1 are what interest us. When *d* = 1.25, the immune system is sufficiently effective to suppress tumor growth without treatment intervention. In contrast, when *d* = 1 or less, then without DC vaccination, the tumor grows rapidly. However, if the tumor challenge has been preceded by two doses of a DC vaccine, then even when *d* = 1, tumor growth is suppressed. We also extended the simulations out 165 days after the tumor challenge (not pictured). For *d* ≥ 1, the tumor shrank to zero after vaccination and did not regrow. Numerically, this indicates that the zero tumor equilibrium is stable.

## 5 Future Directions

In this paper, we presented a model of Dendritic Cell trafficking and interaction with a tumor cell population. With this model, we achieved simulation outcomes that quantitatively match published data from studies on mice (Lee et al., 2007) that first were challenged with tumor and subsequently treated with DC therapy. We then used the model to test a wider variety of hypothetical treatment scenarios. In addition, we examined the effects of prophylactic vaccination with DCs. The simulation results from the prophylactic vaccination scenarios that were discussed in the previous section are preliminary, but do show qualitative agreement with a different set of data from published laboratory experiments on mice (Preynat-Seauve et al., 2007).

In future work, we will investigate how to scale this model to reflect tumor growth and DC trafficking in humans. This will involve a careful examination of the effect of distances between the tumor site and the lymph organs. In addition, we will further investigate the effects of prophylactic vaccination. Our goal is to determine which parameters and model terms need tuning to achieve quantitative as well as qualitative outcomes that reflect the laboratory data.

## Conflict of Interest Statement

The authors declare that the research was conducted in the absence of any commercial or financial relationships that could be construed as a potential conflict of interest.

## Appendix

### A Parameter Values

The parameters are described in Table A1. Refer to Ludewig et al. (2004) for justification and confidence intervals for the parameters measured in that study.

**Table A1:** **Parameter values**.

Parameter name	Description	Value	Units	Reference
^*a_D_*^	Natural death rate of DCs	0.2310	1/day	Ludewig et al. (2004)
$a_{E_{a}S}$	Death rate of activated CTLs in spleen	0.1199	1/day	Ludewig et al. (2004)
$a_{E_{a}T}$	Death rate of activated CTLs in tumor compartment	0.462	1/day	de Pillis et al. (2006)
$a_{E_{m}}$	Natural death rate of memory CTLs	0.01	1/day	Ludewig et al. (2004)
*α*	Component of *μ_BTE_*	1	Cell	
$b_{a}={\tilde{b}}_{a}∕Q_{spleen}$	Per cell activation rate of memory CTLs by DCs	0.01	1/(cell · day)	
${\tilde{b}}_{a}$	Activation rate of memory CTL concentration by DCs	1 × 10^−3^	ml/(cell · day)	Ludewig et al. (2004)
$b_{DE}={\tilde{b}}_{DE}∕Q_{spleen}$	Per cell elimination rate of DCs by activated CTLs	1.3 × 10^−6^	1/cell · day	
${\tilde{b}}_{DE}$	Elimination rate of DCs by activated CTLs (per concentration)	1.3 × 10^−7^	ml/cell/day	Ludewig et al. (2004)
*b_p_*	Maximal expansion factor of activated CTL	85	1/day	Ludewig et al. (2004)
*c*	Rate at which activated CTLs are inactivated by tumor cells	9.42 × 10^−12^	1/(cell · day)	de Pillis et al. (2006)
*d*	Maximum fractional tumor kill by CTLs	0.35	1/day	Fit to Lee et al. (2007)
*E_naive_*	Number of naive CTL cells contributing to primary clonal expansion	370	Cells	Ludewig et al. (2004)
*k*	Carrying capacity of tumor	1.0 × 10^9^	Cells	Fit to Lee et al. (2007)
*l*	Immune strength scaling exponent	$\frac{2}{3}$	Unitless	*Ad hoc* value
*m*	Maximum recruitment rate of DCs to tumor site	2.4388 × 10^4^	Cells/day	Fit to Lee et al. (2007)
*μ_B_*	Rate of DC emigration from blood. Note: *μ_B_* = *μ_BS_* + 24(*μ_BLi_* + *μ_BLu_* + *μ_BO_*), the sum of DC outflow to the spleen, liver, lung, and other parts of the body	27.072	1/day	Ludewig et al. (2004)
$\mu_{BB}={\tilde{\mu }}_{BB}-\mu_{BL}$	Scaled and shifted elimination (clearance and extravasation) rate of CTL from blood	5.7	1/day	
${\tilde{\mu }}_{BB}$	Total elimination rate of CTL from blood	5.8	1/day	Ludewig et al. (2004)
*μBL*	Transfer rate of DCs from the blood to the liver	0.1	1/day	Ludewig et al. (2004)
*μBS*	Transfer rate of DCs from blood to spleen	2.832	1/day	Ludewig et al. (2004)
$\mu_{BSE}={\tilde{\mu }}_{BSE}Q_{spleen}∕Q_{blood}$	Scaled transfer rate of activated CTLs from blood to spleen	7.33 × 10^−4^	1/day	
${\tilde{\mu }}_{BSE}$	Transfer rate of activated CTLs from blood to spleen	0.022	1/day	Ludewig et al. (2004)
$\mu_{BTE}=\mu_{BB}\frac{T}{\alpha +T}$	T-dependent rate at which effector cells enter the tumor compartment from the blood	Calculated	1/day	
*μ_LB_*	Transfer rate of DCs from the liver to the blood	0.51	1/day	Ludewig et al. (2004)
$\mu_{SB}^{Normal}$	Normal DC transfer rate from spleen to blood	0.512	1/day	Ludewig et al. (2004)
$\mu_{SB}^{*}$	DC reduced transfer rate from spleen to blood	0.012	1/day	Ludewig et al. (2004)
*μ_TB_*	Rate of transfer of DC from tumor to blood	0.0011	1/day	*Ad hoc* value
*q*	Value of *T* necessary for half-maximal DC recruitment	100	Cells	Fit to Lee et al. (2007)
*Q_blood_*	Murine blood volume	3	ml	Ludewig et al. (2004)
*Q_liver_*	Murine liver volume	0.5	ml	Ludewig et al. (2004)
*Q_spleen_*	Murine spleen volume	0.1	ml	Ludewig et al. (2004)
*r*	Tumor growth rate	0.3954	1/day	Fit to Lee et al. (2007)
*r_am_*	Reversion rate of activated CTL to memory CTL	0.01	1/day	Ludewig et al. (2004)
*s*	Value of ${\left(E_{tumor}^{a}∕T\right)}^{l}$ necessary for half-maximal activated CTL toxicity	1.4	Unitless	Fit to Lee et al. (2007)
*τ_D_*	Duration of preprogramed CTL divisions	0.5	Days	Fit to Lee et al. (2007)
$\theta_{D}={\tilde{\theta }}_{D}Q_{spleen}$	Scaled threshold in DC density in the spleen for half-maximal proliferation rate of CTL	212	Cell	
${\tilde{\theta }}_{D}$	Threshold in DC density in the spleen for half-maximal proliferation rate of CTL	2.12 × 10^3^	Cell/ml	Ludewig et al. (2004)
$\theta_{shut}={\tilde{\theta }}_{shut}Q_{spleen}$	Scaled threshold in DC density in the spleen for half-maximal transfer rate from spleen to blood	1.3	Cells	Ludewig et al. (2004)
${\tilde{\theta }}_{shut}$	Threshold in DC density in the spleen for half-maximal transfer rate from spleen to blood	13	Cells/ml	Ludewig et al. (2004)

### B Stability Analysis: Jacobians

Recall that *J*_0_ is the Jacobian for the non-delayed system, in other words, its entries are given by *J*_0_(*i*, *j*) = *dF_i_*/*dx_j_*, and *J_τ_*(*i*, *j*) = *dF_i_*/*dz_j_* is the Jacobian for the delayed system. For computational ease, we rename the state variables as follows:

$$\begin{array}{c} D_{blood}=x_{1},\;D_{spleen}\;=\;x_{2},\;E_{blood}^{a}\;=\;x_{3},\\ E_{spleen}^{a}=x_{4},\;E_{blood}^{m}\;=x_{5},\;E_{spleen}^{m}\;=\;x_{6},\\ E_{tumor}^{a}=x_{7},\;T\;=x_{8},\;D_{tumor}\;=\;x_{9}. \end{array}$$

There are only two delayed variables:

$$D_{spleen}\left(t-\tau \right)=z_{2},\;E_{spleen}^{a}\left(t-\tau \right)=z_{4}.$$

We perform the stability analysis without any treatment, i.e., *v_blood_* = *v_tumor_* = 0. The non-zero elements of *J*_0_ are calculated to be:

$$\begin{array}{l} J_{0}\;(1,\;1)=-\mu_{B}\\ J_{0}\;(1,\;9)=\mu_{TB}\\ J_{0}\;(2,\;1)=-\mu_{BS}e^{\left(\frac{-\mu_{BS}x_{1}}{Max\;D}\right)}\\ J_{0}\;(2,\;2)=-a_{D}\;-b_{DE}x_{4}\\ J_{0}\;(2,\;4)=-b_{DE}x_{2}\\ J_{0}\;(3,\;2)=\frac{-\Delta \mu }{\theta_{shut}}\frac{x_{4}}{{\left(1\;+\;x_{2}/\theta_{shut}\right)}^{2}}\\ J_{0}\;(3,\;3)=-\mu_{BB},\\ J_{0}\;(3,\;4)=\mu_{SB}(D_{spleen})\;,\\ J_{0}\;(4,\;2)=\frac{-\Delta \mu }{\theta_{shut}}\frac{x_{4}}{{\left(1\;+\;x_{2}/\theta_{shut}\right)}^{2}}+b_{a}x_{6}\\ J_{0}\;(4,\;3)=\mu_{BSE}\\ J_{0}\;(4,\;4)=\mu_{SB}(D_{spleen})-a_{E_{a}S}-r_{am}\\ J_{0}\;(4,\;6)=b_{a}x_{2}\\ J_{0}\;(5,\;2)=\frac{-\Delta \mu }{\theta_{shut}}\frac{x_{6}}{{\left(1\;+\;x_{2}/\theta_{shut}\right)}^{2}}\\ J_{0}\;(5,\;5)=-\mu_{BB},\;J_{0}\;(5,\;6)=\mu_{SB}(D_{spleen})\;\\ J_{0}\;(6,\;2)=-\left(b_{a}-\frac{\Delta \mu }{\theta_{shut}}\frac{1}{{\left(1\;+\;x_{2}/\theta_{shut}\right)}^{2}}\right)x_{6}\\ J_{0}\;(6,\;4)=r_{am}\\ J_{0}\;(6,\;5)=\mu_{BSE}\\ J_{0}\;(6,\;6)=-(a_{E_{m}}+b_{a}x_{2}+\mu_{SB}(D_{spleen})\;)\\ J_{0}\;(7,\;3)=\mu_{BTE}\;(T)\\ J_{0}\;(7,\;7)=-a_{E_{a}T}-cx_{8}\\ J_{0}\;(7,\;8)=\frac{\mu_{BB}\alpha }{{\left(\alpha +x_{8}\right)}^{2}}x_{3}-cx_{7}\\ J_{0}\;(8,\;7)=\frac{dsl{\left(x_{7}/x_{8}\right)}^{l-1}}{{\left\{s+{\left(x_{7}/x_{8}\right)}^{l}\right\}}^{2}}\\ J_{0}\;(8,\;8)=\;r-\frac{2rx_{8}}{k}-\frac{dsl{\left(x_{7}/x_{8}\right)}^{l}}{{\left\{s+{\left(x_{7}/x_{8}\right)}^{l}\right\}}^{2}}+\frac{d{\left(x_{7}/x_{8}\right)}^{l}}{s+{\left(x_{7}/x_{8}\right)}^{l}}\\ J_{0}\;(9,\;8)=\frac{mq}{{\left(q+x_{8}\right)}^{2}}\\ J_{0}\;(9,\;9)=-(\mu_{TB}+a_{D}) \end{array}$$

where $\mu_{BTE}\left(T\right)=\mu_{B}B\frac{T}{\alpha +T},\;and\;\mu_{SB}\left(D_{spleen}\right)=\mu_{SB}^{*}+\frac{\Delta \mu }{1+D_{spleen}∕\theta_{shut}}$.

There are only two non-zero elements of *J_τ_*:

$$J_{\tau }\left(5,4\right)=b_{p}\frac{z_{5}\theta_{D}}{{\left(\theta_{D}+z_{4}\right)}^{2}},\;J_{\tau }\left(5,5\right)=\frac{b_{p}x_{4}}{\theta_{D}+z_{4}}.$$

As referenced in the text, there are two positive tumor equilibrium values, *T**:

$$T_{1}^{*}\approx 9.72\times 10^{8};\;T_{2}^{*}\approx 9.81\times 10^{8}.$$

However, the first one results in a non-biologically relevant equilibria as it corresponds to a negative equilibrium value for *x*_2_, *x*_4_, *x*_5_. For the non-delay case (i.e., *τ* = 0,) there are no *J_τ_* elements. Thus we consider the eigenvalues of $J_{0}$. At the positive (biologically relevant) equilibria, the eigenvalues of *J*_0,_ as computed via MatLab to four decimal places, are

$$\begin{array}{l} \vec{e}=[-27.0720,-11.1021,-0.6765,-0.4719,-0.3918,\\ \;-0.3092,-0.2321,-5.7000,-5.7000]. \end{array}$$

Each eigenvalue clearly satisfies ℛ(λ) < 0, indicating that, in the non-delay case, the biologically relevant equilibrium is stable.
